# Complete Genome Sequence of the Endophytic Bacterium Bacillus cereus PgBE311, Isolated from *Panax ginseng*

**DOI:** 10.1128/MRA.01382-18

**Published:** 2018-11-29

**Authors:** Chi Eun Hong, Jang Uk Kim, Jung Woo Lee, Kyong Hwan Bang, Ick-Hyun Jo

**Affiliations:** a Department of Herbal Crop Research, National Institute of Horticultural and Herbal Science, Rural Development Administration, Eumseong, Republic of Korea; University of Maryland School of Medicine

## Abstract

Bacillus cereus PgBE311, isolated from the root tissue of a 5-year-old Panax ginseng plant, showed activities against the fungal pathogens Cylindrocarpon destructans and Botrytis cinerea. Here, we report the genome sequence of B. cereus PgBE311. The bacterium contains antibiotic-related gene clusters and has the potential to stimulate plant growth.

## Abstract (precis)

Bacillus cereus PgBE311, isolated from the root tissue of a 5-year-old Panax ginseng plant, showed activities against the fungal pathogens Cylindrocarpon destructans and Botrytis cinerea. Here, we report the genome sequence of B. cereus PgBE311.

## ANNOUNCEMENT

Bacillus cereus is a plant growth-promoting endophytic bacterium that confers resistance against a broad range of phytopathogens by producing versatile metabolites (1). B. cereus strain TG1-6 was reported to be highly salt tolerant and effective in enhancing plant tolerance to drought stress; it also produces the phytohormones auxin and aminocyclopropane-1-carboxylate (ACC) deaminase (2). Another strain, AR156, induces resistance against phytopathogens by triggering induced systemic resistance (3). Five-year-old ginseng plants were harvested from the National Institute of Horticultural and Herbal Science of the Rural Development Administration in Chunbuk Province, Republic of Korea (127°45′13.14″E, 36°56′36.63″N). The root tissues were washed with tap water to remove soil and surface pollutants. Then, the samples were sterilized with 70% ethanol, 12% NaOCl, and sterilized water and homogenized in 1 ml sterilized water using a tissue lyser. Ten microliters of homogenate was spread onto an LB plate at room temperature, and a single isolate of each colony was subcultured at 28°C and 200 rpm for 20 h. Among the isolates, strain PgBE311 was identified as B. cereus based on 16S rRNA gene sequence analysis.

The B. cereus PgBE311 genome was constructed *de novo* using PacBio sequencing data. Genome sequencing was performed by Chunlab, Inc. (Seoul, Republic of Korea). Briefly, bacterial genomic DNA was extracted by incubation with Chelex 100 resin (catalog number 143-2832; Bio-Rad, Hercules, CA, USA) and proteinase K at 65°C for 30 min and boiling at 100°C for 10 min. After centrifugation at 10,000 *× g* for 10 min, the supernatants containing bacterial genomic DNA were obtained. The sequencing libraries were prepared according to the manufacturer’s instructions for 20-kb template preparation with the BluePippin size-selection system (Sage Science, Beverly, MA, USA) using the PacBio DNA template prep kit 1.0 (15-kb size cutoff; Menlo Park, CA, USA). The libraries were quantified using Quant-IT PicoGreen (Invitrogen, Carlsbad, CA, USA) and qualified using a high-sensitivity DNA chip (Agilent Technologies, Waldbronn, Germany). Subsequently, the libraries were sequenced with the PacBio RS II platform using P6-C4 chemistry in an 8-well single-molecule real-time (SMRT) cell v3. Filtering was performed by the Hierarchical Genome Assembly Process version 2 (HGAP 2) protocol (4) with default parameters. Assembly was performed using the HGAP 2 protocol with default parameters in SMRT Analysis version 2.3.0. A total of 86,335 paired reads were generated from the library. The completely assembled genome sequence of B. cereus PgBE311 contained 5,744,144 bp with a GC content of 32.3% and three contigs with lengths of 5,230,609 bp, 498,729 bp, and 14,806 bp. The depth of coverage was 223.72-fold. Gene finding and annotation were performed using the EzBioCloud genome database and Rapid Annotation using Subsystem Technology (RAST) server (5). The protein-coding sequences (CDSs) were predicted by Prodigal version 2.6.2 (6) and classified into groups based on their roles, with reference to orthologous groups (eggNOG version 4.5, http://eggnogdb.embl.de) (7). A total of 5,625 CDSs, 109 tRNA genes, and 42 rRNA genes were identified.

Amino acids and derivative CDSs were the most common subsystems of B. cereus PgBE311, as determined by RAST analysis. We identified useful CDSs that have the potential to promote plant growth and biodegradation and produce antibiotics. B. subtilis and Bacillus mojavensis are known to produce cell wall-degrading enzymes and lipopeptides, including iturins, fengycins, and surfactins (8). Furthermore, lipopeptides contribute to inhibiting the growth of fungal pathogens. B. cereus PgBE311 also harbored plipastatin synthase subunit and 4′-phosphopantetheinyl transferase ffp, which are involved in the production of lipopeptides. The CDSs producing siderophores, such as the petrobactin-mediated iron uptake system, bacillibactin siderophore, siderophore assembly kit, and siderophore anthrachelin, were detected. A siderophore is a high-affinity iron-chelating compound secreted by microbes that can stimulate plant growth (9). Based on these analyses, B. cereus PgBE311 may have antipathogenic activity and promote plant growth. Additionally, we found that B. cereus PgBE311 contained the BenK transporter and other elements of the benzoate degradation pathway, which formed a cluster with aromatic amino acid biosynthesis genes (10). This pathway may be useful for enhancing bioremediation by degrading environmental pollutants harboring aromatic compounds.

Our genome data will help us understand the genetic and functional characteristics of PgBE311 and its potential use as a biocontrol agent.

### Data availability.

The genome sequence of B. cereus PgBE311 was deposited in the NCBI Sequence Read Archive (SRA) under the SRA accession number SRR7967474 and the BioProject number PRJNA485192.
